# Could a Focus on the “Why” of Taxonomy Help Taxonomy Better Respond to the Needs of Science and Society?

**DOI:** 10.3389/fmicb.2022.887310

**Published:** 2022-05-19

**Authors:** Leighton Pritchard, C. Titus Brown, Bailey Harrington, Lenwood S. Heath, N. Tessa Pierce-Ward, Boris A. Vinatzer

**Affiliations:** ^1^Strathclyde Institute for Pharmacy and Biomedical Sciences (SIPBS), University of Strathclyde, Glasgow, United Kingdom; ^2^Department of Population Health and Reproduction, University of California, Davis, Davis, CA, United States; ^3^Department of Computer Science, Virginia Tech, Blacksburg, VA, United States; ^4^School of Plant and Environmental Sciences, Virginia Tech, Blacksburg, VA, United States

**Keywords:** taxonomy, genomics, identification, prokaryotes, phylogeny, taxonomic ranks, species

## Abstract

Genomics has put prokaryotic rank-based taxonomy on a solid phylogenetic foundation. However, most taxonomic ranks were set long before the advent of DNA sequencing and genomics. In this concept paper, we thus ask the following question: should prokaryotic classification schemes besides the current phylum-to-species ranks be explored, developed, and incorporated into scientific discourse? Could such alternative schemes provide better solutions to the basic need of science and society for which taxonomy was developed, namely, precise and meaningful identification? A neutral genome-similarity based framework is then described that could allow alternative classification schemes to be explored, compared, and translated into each other without having to choose only one as the gold standard. Classification schemes could thus continue to evolve and be selected according to their benefits and based on how well they fulfill the need for prokaryotic identification.

## The *Why* of Taxonomy

In an insightful article in 2021 in the International Society of Microbial Ecology Journal, Hugenholtz and colleagues provided a comprehensive review of the history of prokaryotic taxonomy and highlighted current and future challenges (Hugenholtz et al., 2021). The article contributed rich context for the ongoing debate over taxonomy and nomenclature, in particular, in regard to the uncultured majority of prokaryotes (Rinke et al., 2013), and built a strong argument for genome-based taxonomy. However, we believe that improving taxonomy using genomics should not stop us from more fundamentally rethinking both its structure and applications and answering the question of *why* we practice taxonomy in the first place.

The *why* of taxonomy becomes clear when we consider all three elements of taxonomy: classification, nomenclature, and *identification* (Cowan, 1965). Importantly, only when taxonomy permits identification of an unknown as a member of a named group, a taxon, with characteristics that are distinct and relevant (and, therefore, predicts that the unknown has these same distinct and relevant characteristics), can it answer to scientific and societal needs. In this context, classification becomes a prerequisite for meaningful identification by creating clear and distinct boundaries of practical relevance between groups of microbes. The need for nomenclature also follows from identification as unambiguous naming is critical for effective communication, and possibly societal action (e.g., selection of the appropriate clinical treatment), following identification.

Here are some examples of what we intend by meaningful identification of microbes. In basic science, identification may simply consist in finding the position of an organism in a phylogenetic tree to reveal its evolutionary relationship to other organisms. Beyond single organisms, reliable identification of community members may delineate community structure to understand system-level responses central to the environmental roles of microbes. From a societal and policy-making perspective, meaningful identification of an unknown as a member of a taxon may predict a threat, such as the potential to cause disease, and may trigger regulatory action under national or international laws, such as import/export restrictions or implementation of quarantine or isolation. On the other hand, identification as a member of a group with beneficial characteristics, may lead to intellectual property protection. Therefore, from the perspective of *identification*, it becomes clear that taxonomy answers important needs in both basic science and society.

## Questioning the Current Practice of Taxonomy

When we focus on *identification*, aspects of current taxonomy that some taxonomists may consider to be unchangeable tenets and needs of taxonomy can be seen to serve only the *how* of the current practice of taxonomy, but not the *why* of taxonomy itself. In our view, several elements of classical taxonomy can be reassessed on these grounds, such as: the reliance on historic and subjective taxonomic ranks when a different number and distribution of ranks might be more useful; making a distinction between “taxonomy” and “strain typing” when the boundary between the two is arguably subjective; considering species and subspecies to be the smallest recognizable taxonomic units when the possession of laterally-transferred functional regions, such as pathogenicity islands, may be a more appropriate reason for demarcating a group of isolates; requiring name-bearing type strains to describe species when the vast majority of prokaryotes are likely fastidious or otherwise practically unculturable; the attachment to Latin binomials which draw attention to a limited set of properties; and the insistence on a single scheme of stable and unique hierarchical names to describe a collection of items that could be partitioned usefully in many other ways. In the current era of databases that can provide persistent stable IDs to individuals, we believe it is time for microbiology to adopt the advantages that technology brings to data organization, while preserving the best features of classical taxonomy.

Some of the assumptions relating to genome-based taxonomy can also be questioned if we consider the *why* of taxonomy. For example, an assumption that phylogenetic clades based on the alignment of core genes or proteins should be the only basis for the circumscription of named groups prioritizes vertical transfer of genomic information. However, in many societally-important circumstances, such as antimicrobial resistance or the presence of pathogenicity islands, the phenotype of interest may be governed by horizontally-transferred genes (Soucy et al., 2015). In these circumstances, a classification that prioritizes the phenotype and considers lateral transfer of genomic information might be more useful. Such a classification might be better facilitated by a system of individual genome accessions with flexible labelling (like tags in a Google Mail inbox) than a hierarchical naming scheme. Hugenholtz et al. (2021) argue that hierarchical taxonomic ranks and binomial species are a necessity because of biologists’ reluctance to take up new systems, such as the rank-free PhyloCode (Cantino and de Queiroz, 2020). However, reluctance to change is not an argument against the utility of change and, in any case, might very well apply more to taxonomists than to biologists overall.

Nonetheless, there are challenges inherent to taxonomy that cannot be avoided. Circumscription of existing groups requires revision as new knowledge becomes available, and such reclassification necessarily requires translation from one named taxon to another, for example, when reading literature about the same organism before and after reclassification occurred. Also, there is more than one reasonable hierarchical classification system. For this reason, and because we do not have perfect knowledge and understanding of the hierarchical process of evolution, it is inevitable that no single human-created model will capture all useful categorizations of organisms—regardless of the claim that “biologists now agree that taxonomy should be based on evolutionary relationships as the most natural way of arranging organisms” (Hugenholtz et al., 2021).

## Where to go From Here?

If we accept that our current taxonomic system is conditioned by history and just one of many reasonable choices, perhaps we could dare to try some unconventional solutions to answer the *why* of taxonomy within the landscape of genomic data. For example, what if we acknowledged that the distinction between named species and subspecies on one hand and informal within-species clades, clonal complexes, and outbreak strains on the other hand is subjective and these taxa could all be handled using a single set of rules? Would it help us understand biology better if we were to give species complexes, within-species clades, and other monophyletic groups, the same importance as current taxonomic ranks, as proposed in the PhyloCode (Cantino and de Queiroz, 2020)? What if we could use a neutral framework of genome identifiers to explore and compare new ways to infer evolutionary relationships between organisms, beyond core gene phylogenies? What value could be gained from approaches that combine genome similarity with similarity in gene content reflecting adaptation to different ecological niches? Is it possible to construct complementary phylogenetic or even non-phylogenetic classification systems, similar to library cataloging systems, such as the subject-based Dewey system? Specialized schemes could be based on the content of functional classes of genes (corresponding to subjects in the Dewey system), such as pathogenicity/virulence genes that are important in a biosecurity context, where the risk is governed by gene content more than evolutionary relationship.

To implement, compare, and perhaps unify these alternative classification schemes, we would need a “Rosetta Stone” to translate between them. We are thus building a “genomic coordinate system” as part of the *genomeRxiv* platform using the Life Identification Number (LIN) approach (Vinatzer et al., 2017; Tian et al., 2020) analogous to a map grid reference, which hierarchically subdivides and labels the entire prokaryotic genome space into uniquely-labelled volumes (or voxels) of sequence-similar genomes that, at their finest resolution, contain a single genome uniquely identified with its “coordinate” (its LIN). We believe that this is a practical, quantitative, automatable, stable, and robust solution to the problem of translating among classification schemes, for example, between validly published prokaryotic named species and genome-based species clusters (Sanford et al., 2021). More in general, classifications of prokaryote genomes made by one scheme, such as descent from a common ancestor, or “*bona fide* species definitions,” can then be expressed as combinations of uniquely-labelled voxels and compared to similar combinations obtained by alternative classification schemes, such as presence or absence of specific genes. For example, besides assigning plant growth promoting bacteria to named species based on common ancestry, they could also be assigned to a taxon called “plant growth promoters.” When additional bacteria are found to promote plant growth, they could be added to this function-based taxon. The LINs of the genomes reclassified as “plant growth promoters” however would not change, maintaining stability.

The LIN-based coordinate scheme is hierarchical, but purely descriptive. It neither requires nor imposes an evolutionary model. It is neutral on the questions of nomenclature and classification. It requires no consensus on a single scheme but instead enables meaningful translation among alternative schemes. For example, the validly published species names, informal phylotypes, and population clusters within the *Ralstonia solanacearum* species complex were all circumscribed using LINs allowing the identification of any newly sequenced genome as a member of each of these taxa, simultaneously (Sharma et al., 2022). Our proposal is also by nature able to accommodate the many, as yet unknown and unclassified, prokaryotes whose sheer number currently poses a significant problem for nomenclature and classification. So long as a genome sequence is available, a coordinate in genome space can be automatically assigned and used as an identifier even before the genome is classified as a member of an already described, or still to be named, taxonomic group. If this genome is of an emerging pathogen, the identifier can be used for clear communication about an ongoing disease outbreak from the moment the genome has been sequenced without having to wait for a validly published name. An example from virology is that one cannot search for the earliest reports of SARS-CoV-2 by searching for “SARS-CoV-2” since scientists referred to it as “2019-nCoV” until the Coronaviridae study group of the International Committee on Taxonomy of Viruses decided on its name (Coronaviridae Study Group of the International Committee on Taxonomy of Viruses, 2020). A LIN-based identifier for the first SARS-CoV-2 genome, as we propose, may have enabled ready tracing of SARS-CoV-2 genomes, without the confusion imposed by nomenclature changes.

In conclusion, we propose to treat genome sequence data neutrally to build a genotypic framework. We do not propose to privilege a specific set of genes, or a specific evolutionary model or reconstruction method as an immutable truth, or “gold standard,” against which all other schemes would be measured. Instead, we propose a whole-genome framework on which alternative choices of phylogenetic and phenotypic classification schemes can be compared. Like a coordinate system on a map, this framework provides an address for each genome and links to any classification information within any taxonomic system. We expect this framework to provide a landscape on which classification systems that best respond to the needs of science and society can continue to be developed based on the latest biological, biotechnological, and ecological discoveries.

## Data Availability Statement

The original contributions presented in the study are included in the article/supplementary material; further inquiries can be directed to the corresponding author.

## Author Contributions

LP, CB, BH, LH, NP-W, and BV: concept development and writing. All authors contributed to the article and approved the submitted version.

## Funding

Development of the *genomeRxiv* platform is supported by the United States National Science Foundation (DBI-2018522) and the United Kingdom Biotechnology and Biological Sciences Research Council (BB/V010417/1). The funders have no role in study design, data collection and analysis, decision to publish, or preparation of the manuscript.

## Conflict of Interest

*Life Identification Number* and *LIN* are registered trademarks of this Genomic Life Inc. LH and BV report in accordance with Virginia Tech policies and procedures and their ethical obligation as researchers, that they have a financial interest in this Genomic Life Inc. that may be affected by the publication of this manuscript. They have disclosed those interests fully to Virginia Tech, and they have in place an approved plan for managing any potential conflicts arising from this relationship.

The remaining authors declare that the research was conducted in the absence of any commercial or financial relationships that could be construed as a potential conflict of interest.

## Publisher’s Note

All claims expressed in this article are solely those of the authors and do not necessarily represent those of their affiliated organizations, or those of the publisher, the editors and the reviewers. Any product that may be evaluated in this article, or claim that may be made by its manufacturer, is not guaranteed or endorsed by the publisher.
